# Comparative Analysis of Exosomes and Extracellular Microvesicles in Healing Pathways: Insights for Advancing Regenerative Therapies

**DOI:** 10.3390/molecules29153681

**Published:** 2024-08-03

**Authors:** Mikołaj Sędzik, Katarzyna Rakoczy, Jakub Sleziak, Michał Kisiel, Karolina Kraska, Jakub Rubin, Wiktoria Łuniewska, Anna Choromańska

**Affiliations:** 1 Faculty of Medicine, Wroclaw Medical University, 50-367 Wroclaw, Poland; mikolaj.sedzik@student.umw.edu.pl (M.S.); katarzyna.rakoczy@student.umw.edu.pl (K.R.); jakub.sleziak@student.umw.edu.pl (J.S.); michal.kisiel@student.umw.edu.pl (M.K.); karolina.kraska@student.umw.edu.pl (K.K.); jakub.rubin@student.umw.edu.pl (J.R.); wiktoria.luniewska@student.umw.edu.pl (W.Ł.); 2 Department of Molecular and Cellular Biology, Faculty of Pharmacy, Wroclaw Medical University, Borowska 211A, 50-556 Wroclaw, Poland

**Keywords:** exosomes, extracellular vesicles, wound healing

## Abstract

Exosomes and microvesicles bear great potential to broaden therapeutic options in the clinical context. They differ in genesis, size, cargo, and composition despite their similarities. They were identified as participating in various processes such as angiogenesis, cell migration, and intracellular communication. Additionally, they are characterized by their natural biocompatibility. Therefore, researchers concluded that they could serve as a novel curative method capable of achieving unprecedented results. Indeed, in experiments, they proved remarkably efficient in enhancing wound regeneration and mitigating inflammation. Despite immense advancements in research on exosomes and microvesicles, the time for their large-scale application is yet to come. This article aims to gather and analyze current knowledge on those promising particles, their characteristics, and their potential clinical implementations.

## 1. Introduction

Extracellular vesicles (EVs) constitute a differentiated group of phospholipid bilayer-surrounded particles involved in homeostasis maintenance and pathological pathways [1]. The vast majority of cells release EVs to mediate intercellular communication or dispose of redundant molecules [1,2].

The diversity of EVs expresses itself in their size, which ranges from 30 to 1000 nanometers in diameter, as well as their biogenesis, cargo, release mechanisms, and composition of membrane surface [2,3]. Thus, EVs, based on their biogenesis, are classified into three main populations: exosomes (EXOs), microvesicles (MVs), and apoptotic bodies (ABs); however, many new subtypes are continuously added with time [4,5]. The smallest EXOs have a topology identical to the cell, augmented with particular macromolecules, such as lipids, proteins, or glycoconjugates [6]. The biogenesis and composition of EVs will be described later in the article. 

When released into the extracellular space, EVs can influence cells, both placed nearby and distant, in many ways. To do so, they must be adequately recognized by the target cells. Thus, the cells and the EVs present specific molecules on their surfaces. Once recognized by the recipient cell, EVs can influence its functioning by direct ligand–receptor binding process, resulting in surface receptor stimulation, or by releasing transported cargo directly into the cytosol [7]. The described processes influence many processes, including cell differentiation and proliferation, angiogenesis, response to stress, and intercellular communication [2]. EVs are characterized by natural biocompatibility, low toxicity, and immunogenicity. These properties constitute perfect nanoparticles for therapeutic application, especially in regenerative medicine [8]. In recent years, EVs derived from stem or progenitor cells have been considered more and more promising in significant acceleration of wound healing, resulting in both local and systemic EV administration. According to the newest research, treatment based on EVs derived from mesenchymal stem cells (MSC) can visibly promote the regeneration of vessels, nerves, and hair follicles and inhibit scar formations. They affect the diversity of wound-healing constituent processes, such as cell migration and proliferation, ECM production, macrophage polarization, and angiogenesis [1,2,8]. EVs can affect a multitude of signaling pathways, such as PI3K/Akt, Wnt, NF-κB, MAPK, VEGF, and TGF-β, which results in the downregulation of inflammation, oxidative stress, and reduction, as well as re-epithelialization induction. Nonetheless, stem cells not only are able to be donors of regenerative EVs, as there are examples in the literature showing that EVs derived from platelets, fibroblasts, macrophages, or even tumor cells are able to achieve great healing results as well. 

Therapies based on EVs have promising perspectives in regenerative medicine; however, clear differentiation between applications of EXOs and MVs is needed. This review aims to compare healing pathways mediated by these two subtypes of EVs, regarding their prospects in clinical applications.

The investigation into the role of EXOs and EVs in healing pathways has captured the attention of numerous research groups. We embarked on electronic searches to uncover relevant literature, utilizing PubMed as our primary indexing database due to its extensive coverage of articles pertinent to our inquiry. Concurrently, we explored Scopus and Web of Science databases to validate and complement our findings. The analysis was performed in the second quarter of 2024. Our search strategy was anchored around key terms: exosomes, extracellular vesicles, and wound healing. This procedure allowed us to find 652 results. The data were narrowed down to articles focused solely on the biology of EVs, specific mechanisms that contribute to the healing process, and possible clinical impact.

## 2. Biogenesis and Composition of Exosomes and Microvesicles

**Figure 1:**
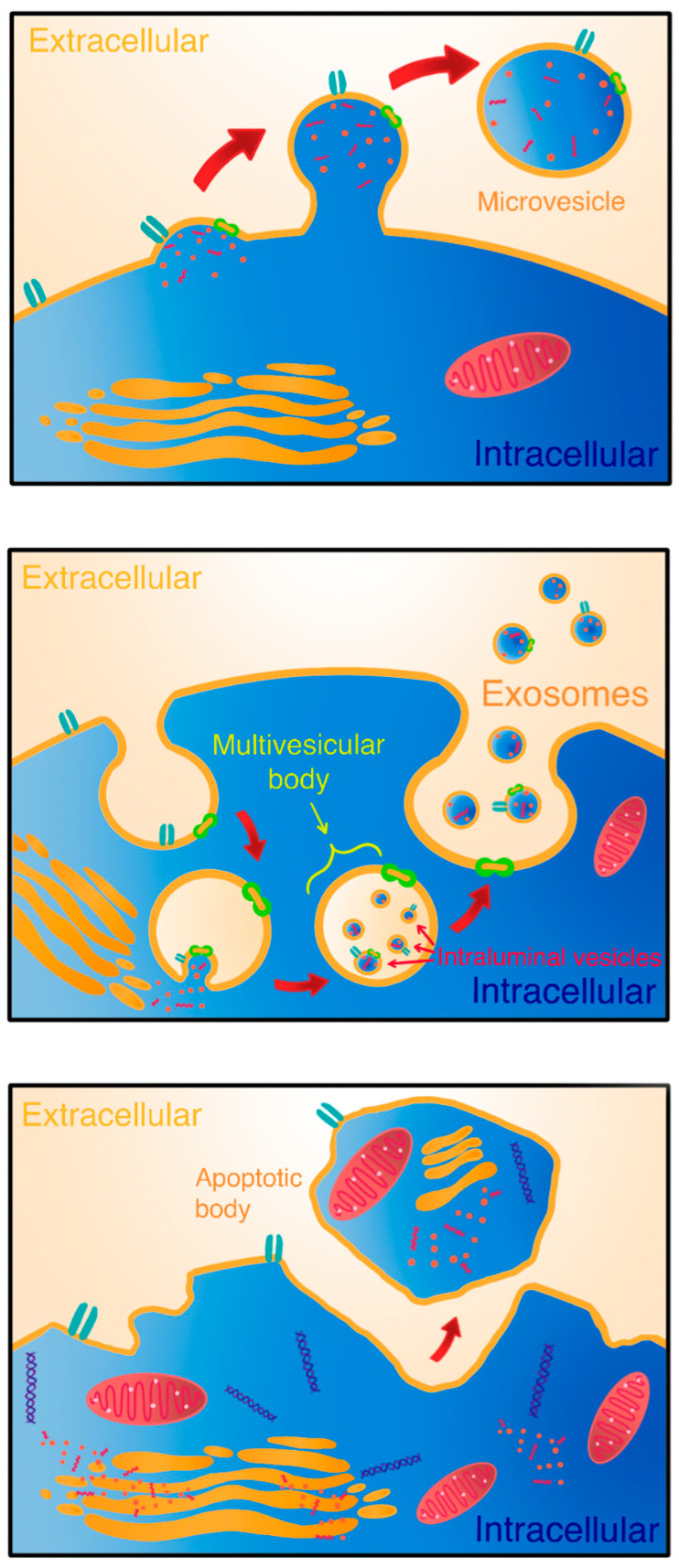
Simplified biogenesis of extracellular vesicles. Microvesicles are directly secreted into the ECM through outward budding of the plasma membrane [16]. In contrast, exosome formation begins within multivesicular bodies, where intraluminal vesicles are generated by the invagination of the endosomal membrane [17]. Apoptotic bodies result from the fragmentation of cells during programmed cell death [18].

EXOs are derived from multivesicular bodies (MVBs) that are formed during the maturation of early endosomes. EXOs form as intraluminal vesicles (ILVs) as a result of the inward budding of the membrane of MVBs and incorporating various cargo (Figure 1). EXOs are then either sent to lysosomes to be degraded or released into extracellular space from within MVBs after MVBs fuse with the cellular membrane [9,10,11,12]. ILV formation can be divided into two phases: first—involving the organization of the endosome membrane into tetraspanin-enriched microdomains, and second—involving the endosomal sorting complex required for transport (ESCRT). The function of ESCRT is thought to be regulating accumulation and sorting cargo loaded into ILVs. ESCRT-independent has also been reported, which is mostly regulated by sphingomyelinases, phospholipase D2 and ARF6 [10,11].

MVs, also called ectosomes, are created by the outward budding of cellular membranes as a result of cytoskeletal protein contraction due to cellular membrane bending at tight spaces or overcrowding at the cell periphery [9,13]. The process is regulated by aminophospholipid translocases such as flippases and scramblases that mediate phospholipid rearrangement and RhoA and Rho-associated coiled-coil-containing protein kinase (ROCK) and LIM Kinase (LIMK) that mediate actin dynamics [7].

EXOs composition comprises of proteins, nucleic acids, such as ssDNA, dsDNA, various RNAs (mRNAs, miRNAs, long non-coding RNAs, tRNAs), mitochondrial DNAs, cytokines, and transcription factor receptors [9,14]. Protein cargo can be divided into cell-specific, which differ between donor cells, i.e., CD45 and MCH-II proteins, and non-specific, which is common in all EXO types [14]. Examples of non-specific proteins are ALIX, tumor susceptibility gene 101, Rab proteins, heat shock proteins, tetraspanins, CD63, CD81, and integrins [11,12].

MVs composition comprises proteins such as membrane proteins (tetraspanins, receptors, ligands, or adhesion molecules), cytoskeletal proteins, heat shock proteins, integrins, and proteins containing post-translational modifications (glycosylation and phosphorylation) [12,15]. MVs can also transport DNA, mRNA, miRNA, and other non-coding RNAs [11,13].

## 3. Role of Exosomes in Healing Pathways

In general, wound healing and tissue regeneration encompass four interconnected phases: hemostasis, inflammation, proliferation (involving re-epithelialization, angiogenesis, and extracellular matrix formation), and tissue remodeling. These sequential phases, when executed as programmed, play a crucial role in restoring homeostasis post-injury. However, various factors such as ischemia, diabetes mellitus, venous stasis disease, or pressure can disrupt this process, increasing the likelihood of dysregulated and pathological inflammation [19]. This altered inflammatory state is characterized by excessive infiltration of neutrophils and macrophages, leading to heightened production of reactive oxygen species (ROS) and inflammatory cytokines. Consequently, mitosis may be impeded, hampering proliferation and migration necessary for granulation tissue formation and epithelialization, thus resulting in the development of non-healing chronic wounds [20,21]. 

Utilization of EXOs presents a potential solution to this problem. EXOs, once regarded as “cellular trash bags”, have emerged as promising agents for inducing healing processes [22]. As mentioned above, EXOs are released by the vast majority of cells in terms of communication. It is noteworthy that an EXO’s origin determines its utility and not every cell can elicit regenerative processes. Study groups focus mainly on EXOs derived from stem cells; however, EXOs derived from platelet-rich plasma (PRP), fibroblasts, or M2-macrophages are able to achieve great healing results as well [21,23,24,25].

EXOs exhibit the capability to mitigate inflammation and ROS production while concurrently promoting angiogenesis and re-epithelialization, thereby facilitating tissue regeneration. These EVs contain a wide array of protein and RNA molecules, which are able to impact all these processes [26]. As mentioned above, chronic inflammatory conditions pose a significant impediment to tissue recovery. EXOs exert influence over the inflammation phase by orchestrating macrophage polarization from the classical M1 phenotype to the alternative M2 phenotype. M1-macrophages are recognized as pro-inflammatory cells that impede tissue repair through the release of IL-1β and TNF-α. It has been shown that elevated levels of M1-macrophages have been linked to increased cellular apoptosis, reduced proliferation, and enhanced fibroblast-mediated scar tissue formation [27,28]. As Lou et al. stated, “impaired macrophage polarization is one of the main causes of healing stagnation in the inflammatory phase and poor wound healing” [29]; therefore, maintaining a proper balance in macrophage polarization is imperative for tissue repair and homeostasis [30,31]. On the other hand, M2-macrophages contribute to repair and wound closure by secreting protective factors such as IL-4 and IL-10 [32]. The role of M2 is to impact surrounding cells through the secretion of the above-mentioned cytokines in order to obtain a “regenerative” environment [33]. For example, Chen et al. found that M2-macrophages secrete EXOs containing high levels of IL-10 mRNA, which then leads to the upregulated expression of IL-10 in cultured cells, resulting in the dominance of regenerative processes [34]. Moreover, M2-macrophages are also able to exert a phenotype switch on M1-macrophages through EXOs [35].

EXOs have been shown to utilize different signaling pathways to achieve macrophage (Mφ) polarization toward the M2 phenotype. The STAT pathway primarily regulates this transition and subsequently enhances the synthesis of anti-inflammatory cytokines [30,36,37]. Quero et al. showed that inhibiting the IL-6/STAT3 and JAK3/STAT3 signaling pathways can lead to the transition of macrophages from an M2 to an M1 phenotype. Additionally, STAT3 can downregulate the pro-inflammatory response by directly inhibiting the NF-κB pathway and potentially through the activation of AKT [30,38]. Ren et al. demonstrated that EXOs containing milk fat globule-epidermal growth factor 8 (MFG-E8) modulated STAT3 axis in macrophages within the post-injury microenvironment, promoting a transition to the M2 phenotype and creating an anti-inflammatory milieu conducive to recovery [32]. Furthermore, miR-203a-3p has been shown by Yang et al. to impact the STAT3 pathway leading to Mφ polarization toward the M2 phenotype. The treatment group observed higher levels of M2 markers (CD206 and ARG-1) in flow cytometry, thicker granulation tissue in hematoxylin and eosin stain (H&E stain), and denser collagen fibers, in contrast to the control group [39]. Moreover, suppression of NF-κB activation has been marked as crucial in polarization toward the M2 phenotype. Interestingly, different molecules carried by EXOs are able to impact the axis. For example, Zhou et al. have observed that bone mesenchymal stem cells transferring miR-146a-5p facilitated M2 polarization through suppressing the TRAF6/NF-κB pathway, eventually resulting in improved diabetic wound healing [40]. Li et al. observed similar results in terms of M2 polarization while using miR-181c. The molecule directly prevents an inflammatory response by binding to TLR4, thereby there is a decrease in the number of white blood cells and respective proinflammatory cytokines levels 24 h after administration. [41]. Also, miRNA let-7b molecules carried by LPS pre-EXOs have been proven to augment wound healing by targeting TLR4 genes, attenuating NF-κB activity, and regulating the inflammatory response [30].

Another signaling pathway described in the literature was the TGFβ axis. In vitro and in vivo research by Wang et al. has shown that ESC-EXOs containing high amounts of RNA cargo, mainly miRNA snRNAs, are able to change Mφ polarization via activation of the TGFβ signaling pathway, thus achieving regenerative abilities. The research group observed a higher expression of M2-macrophage markers such as CD206 and YM1 in rat models treated with ESCs-EXOs, whereas inducible nitric oxide synthase expression, associated with the classical (M1) phenotype, was downregulated. Moreover, H&E stain showed lower numbers of inflammatory cells [25].

As previously mentioned, the augmentation of the proliferation and remodeling phase is a crucial aspect of expediting tissue repair, and EXOs have been demonstrated to possess this capability by modulating various cellular pathways. These are vital steps of the whole process; otherwise, it might lead to incomplete regeneration and scar formation. A study conducted by Dalirfardouei et al. revealed that EXOs derived from menstrual blood-derived mesenchymal stem cells (MenSCs) can enhance angiogenesis through the VEGF pathway. In mice treated with EXOs, VEGF expression was upregulated at day 7 post-wounding, along with increased microvessel density and the presence of CD34-positive cells in immunohistochemistry tests [42]. Another investigation by Huang et al. demonstrated that exosome-mediated VEGF activation led to improved wound healing in patients with type 1 diabetes [43]. Furthermore, PI3K/AKT, Erk, and Wnt/β-catenin pathway pathways seem to be also vital. Li et al. have found that EXOs carrying miR-126-3p might augment blood vessel formation, resulting in accelerated and more complete wound closure after 14 days compared to the control group [44]. Similar results with miR-126-3p have been reported by Ma et al. The study group found that the molecule delivered by ADSC-derived EXOs impacted not only better fibroblast proliferation and migration but also angiogenic processes leading to recovering skin parameters after wounding [45]. Also, bFGF, PDGFBB, and TGF-β carried by EXOs derived from platelet-rich plasma (PRP) have been observed to improve the proliferation phase by impacting PI3K/Akt and Erk pathways. The group treated with PRP-EXOs exhibited superior outcomes, characterized by a longer epithelium, well-organized collagen fibers, and a higher number of new blood vessels, as indicated by the increased density of CD31-stained cells per mm^2^ at day 14 [21]. Moreover, research by He et al. found that MALAT1—a long non-coding RNA—inside of ADSC-EXOs suppresses miR-124, thus activating the Wnt/β-catenin pathway, which can promote cell proliferation and migration crucial for proper wound healing [46]. Furthermore, protein 14-3-3ζ was found to accelerate wound healing by the Wnt/β-catenin pathway in vivo. The study by Zhang et al. showed that administration of human umbilical cord MSC (hucMSC) exosomal 14-3-3ζ resulted in complete epidermal regeneration without scar formation at 4 weeks after treatment [47]. Better tissue remodeling and hence prevention of scar formation was proved to be elevated after miR-192-5p administration. Li et al. found that this molecule is able to directly target the Smad pathway, which led to accelerated wound closure on day 5, 7, 10, 12, and 14 [48].

## 4. Functions and Contributions of Extracellular Microvesicles in Healing

MVs, similar to EXOs, originate from several types of cells, including dendritic cells, endothelial cells, hepatocytes, and blood cells—erythrocytes, leukocytes, and platelets [49]. Despite the fact that EXOs are mainly mentioned for healing mechanisms, there are reports of the relevance of MVs in healing pathways. 

Similarly to EXOs, MVs exert an impact on inflammatory processes by regulating the polarization of macrophages. As mentioned above, polarization toward M2 has been proven to be crucial for enhancement in tissue recovery. It is necessary because uncontrolled or persistent inflammation can lead to unresolved pathology. It has been suggested that the Mφ phenotype transition might be regulated by prostaglandin E2 (PGE2) via induction of Krüppel-like factor 4 (KLF4) [50]. Thus, potential induction of PGE2 levels might be beneficial in terms of healing, although this process requires further research. Boilard et al. have shown that MVs are able to transport enzymes necessary for PGE2 synthesis, thereby indirectly contributing to polarization toward M2 [51] Furthermore, the STAT signaling axis has been reported to partake in this process. Ma et al. have shown that MVs derived from human tumor cells carrying genomic and mitochondrial DNA are able to activate the pathway leading to Mφ polarization and increase the expression of anti-inflammatory molecules, which is beneficial for tissue recovery [52]. The study by Imam et.al. also described the impact of MVs on differentiation macrophages toward the anti-inflammatory M2-like phenotype. The study group observed that burn wounds treated with MVs decreased levels of IL-6-pro-inflammatory cytokine, characteristic of the M1-like phenotype, which was significantly elevated after wounding. Additionally, MVs in burn tissue upregulated the level of anti-inflammatory cytokine IL-10, leading to improvement in healing via the completion of epithelial layer formation, remodeling, and maturation of collagen fibers in wounds [53].

MVs also have a fundamental role in the proliferation phase of wound healing. MVs are able to impact angiogenesis through several molecular signaling pathways. The study by Deregibus et al. showed that MVs derived from human endothelial progenitors (EPCs) contain mRNA impacting PI3K/AKT, Erk, and eNOS relevant for angiogenesis. Moreover, the incorporation of MVs has also been shown to activate these axes through the interaction of α4- and β1-integrins expressed on MVs surface and endothelial cells [54]. The VEGF signaling pathway mediated by MVs also has an important role in the regeneration of microvessels after injury. A study by Zhang et al. demonstrated that pericyte-derived microvesicles (PCMVs) transfer miRNA-145 and miRNA-132 to vascular smooth muscle cells (VSMCs) and vascular endothelial cells (VECs), respectively [55]. Those miRNAs are associated with the S1P signaling pathway, which regulates endothelial barrier function and angiogenesis by the G protein-coupled receptors S1PR1 and S1PR2. S1PR1 and S1PR2 are expressed in VECs and VSMCs and play a role in the regulation of endothelial cell cytoskeletal structure, migration, capillary-like network formation, and vascular maturation [56,57]. A further role of MVs in the process of angiogenesis is linked with cytokines. Platelet-derived microparticles exerted proangiogenic effects through the transfer of some of them, such as VEGF, FGF-2, and PDGF, and the activation of PI3 kinase, src kinase, and ERK [58]. Prokopi et al. showed that EPC cultures containing platelet MVs have the capacity to stimulate endothelial tube formation in vitro in this mechanism [59]. 

Moreover, MVs have been proven to promote cell proliferation and migration. For example, in the study by Ren et al., MVs from ASCs have promoted the proliferation of human umbilical vein endothelial cells (HUVECs), HaCAT, and fibroblasts, which is confirmed by increased gene expression of proliferative markers, such as cyclin D1, cyclin D2, cyclin A1, and cyclin A2 [60]. Furthermore, levels of the protein MMP9, integrin beta-1 (ITGB1) and CXCL16 in HUVECs were increased weighting in favor of enhanced cell migration. The study by Yan et al. has demonstrated that MVs, derived from pluripotent stem cells, contain miR-16-5p, which enhances burn wound healing by targeting desmoglein 3 (Dsg3), thus activating the p38/MARK pathway. It has been shown that induction of this molecular pathway leads to increased keratinocyte migration, enhanced re-epithelialization, and better-organized collagen fibers in the group treated with MVs [61]. MVs derived from wound myofibroblasts (Wmyos)—cells that differentiate mainly from fibroblasts and appear during the phase of the granulation tissue formation—contain several cytokines, among others, placental growth factor 1 (PLGF-1) [62]. This molecule is a member of the VEGF family and studies have shown that PLGF-1 can directly stimulate the migration of fibroblasts and collagen I production, and thereby might be beneficial in the wound healing process [63]. In the late stage of wound healing, it is crucial to inhibit excessive cell proliferation and thereby prevent scar formation. 

The study by Yang et al. showed that human umbilical cord mesenchymal stem cells (HUCMSC-MVs) or endothelial progenitor-cell-derived extracellular vesicles (EPC-EVs) deliver 14-3-3ζ protein, which regulates the phosphorylation of YAP and its interaction with phosphorylated-LATS. The phosphorylation of YAP by p-LATS leads to inhibition of the Wnt/β-catenin pathway [64]. Thus, inhibits excessive cell proliferation and collagen deposition, which leads to inhibiting scar formation [65,66].

## 5. Comparative Analysis: Exosomes vs. Extracellular Microvesicles in Healing Pathways

As already described, the mechanism of origin differs between EXOs and MVs, since they are a product of endocytosis and budding, respectively. Although both mentioned EV subtypes bear resemblance in the form of a similar lipid bilayer membrane and cargo, different biogenic pathways lead to some varying contents. Mainly, EXOs contain extracellular matrix proteins, whereas MVs consist of membrane and cytoplasmic proteins [67,68]. In a 2021 study by Guan et al., a total of 112 differential proteins and 50 differential metabolites were found between EXOs and MVs. The research established that 20 up-regulated proteins in EXOs were related to such processes as cell surface receptor signaling pathway, mitotic cytokinesis, and positive regulation of GTPase activity. However, the 92 upregulated proteins in MVs were primarily associated with complement activation, receptor-mediated endocytosis, and immune response [69]. Nonetheless, both vesicles share the CD47 protein on their surface, which allows them to avoid phagocytosis and extend circulation time as a result [70,71]. Furthermore, EXOs and MVs share CD9 and CD63 markers, yet Syntenin-1 is found only in EXOs, whereas Actinin-4 is found solely in MVs [69,70].

A notable difference between EXOs and MVs is their size, where the latter are larger, which may result in their capability to carry more kinds of protein [72]. However, contrary to other research, a 2021 work by D’Souza et al. has proved otherwise [73]. On the other hand, a smaller size of EXOs seems to be favorable if cellular uptake and eliciting cellular response are concerned [74]. Moreover, bioavailability after oral administration and stability are also observed, yet some of the research was conducted on liposomes instead of EVs due to their similarity [72,75].

Both EXOs and MVs can be used in order to achieve a desired effect on selected recipient cells by either delivering their native cargo or appropriately loaded drugs. Furthermore, both have the ability to target multiple cells, which stems from their relevant parental cells. It seems as if MVs bear more resemblance to their parental cells than EXOs, which might be a characteristic worth noting when choosing the right vessel. Nevertheless, both EV subtypes can either provoke immunostimulation or immunosuppression, as predestined by their source [76]. Both particles are able to promote polarization to the M2 macrophage phenotype as well as induce angiogenesis, proliferation, cell migration, and inhibition of scar formation, thus partaking in many processes required for wound healing, as shown in Table 1 and Table 2. Depending on the desired effect, different MSC sources are used, which allows great versatility of both mediums (L Fu, n.d.)(L Fu, n.d.). Despite the known tissue origin, EVs’ features, composition, and functions may present a degree of heterogeneity caused by the genomic state of cells and varying protein concentrations within them, among others. This implies a need for thorough control of EV production to achieve the expected therapeutic impact [77,78]. 

**Figure 2:**
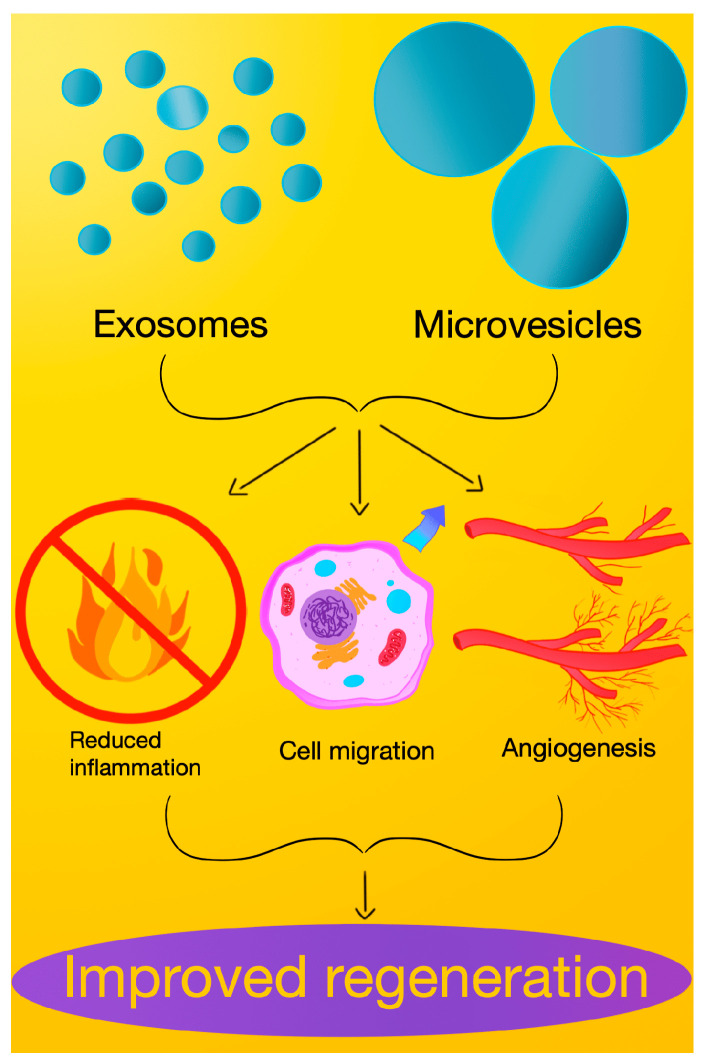
Exosomes and microvesicles improve tissue regeneration via enhanced angiogenesis, induction of cell migration, and reduction in inflammation.

As both particles partake in similar or even the same healing pathways, as shown in Figure 2, few studies have shown a significant difference in function between them. Although EXOs have attracted great scientific interest in recent years, MVs have been successfully trialed in numerous fields. For instance, unlike EXOs, MVs have been shown to increase mitochondrial function in recipients’ brain endothelial cells and can be used to transfer mitochondria to neurons [73].

Apart from therapeutic application, both described EVs have the potential to be used as non-invasive markers in neoplasms as well as neurodegenerative diseases and others [78,79,80].

## 6. Clinical Implications and Future Perspectives

Currently, there are numerous clinical trials focused on the application of EVs, including MVs and EXOs, not only for wound healing but also as biomarkers, cell-free therapeutic agents, drug delivery carriers, and cancer vaccines (Table 3) [81]. However, scaling up the production of EVs is limited by the challenge of their isolation. The most effective methods currently include ultracentrifugation, ultrafiltration, differential centrifugation, size-exclusion chromatography, immunoaffinity, and polymer precipitation. While ultrafiltration shows promise in EXO separation, it faces issues like membrane clogging, which tangential flow filtration may address, but it still needs further investigation [82]. For MVs, the most common isolation method is differential centrifugation, usually involving fewer steps [83].

A significant challenge for the widespread use of EVs in mass production is their low yield under conventional culture conditions and diminishing effectiveness in vivo over time [84]. Recent research has highlighted the impact of preconditioning (priming) methods, which can enhance the biological action of EVs and improve healing outcomes. Preconditioning can be achieved through biophysical cues, such as three-dimensional cultures and electric pulsing, or biochemical methods, including hypoxia, growth factors, enzymes, hormones, and pro-inflammatory agents [84,85].

EVs can be isolated from cells or biofluids, with mesenchymal stem cells (MSCs) being a prominent source due to their robust EV secretion [86]. MSCs can be derived from various sources such as bone marrow, adipose tissue, human umbilical cord, or even menstrual blood [87]. EVs offer advantages in terms of storage, transportation, and cost-effectiveness compared to stem cell therapy, which is time-consuming. Their lipid bilayer structure ensures high bioavailability, allowing them to traverse the circulatory system and penetrate cellular barriers. Additionally, EVs lack major histocompatibility complex (MHC) class I or II proteins, making them low-immunogenic nanocarriers ideal for drug delivery systems, outperforming synthetic carriers like liposomes and nanoparticles [86,87,88,89].

Stem-cell-derived extracellular vesicles (SC-EVs) offer promising regenerative potential, varying with the type of mesenchymal stem cells used. Bone marrow stem-cell-derived EVs (BMSC-EVs) are commonly used in clinical trials. They contain lncRNA H19 and lncRNA KLF3-AS1, which promote fibroblast proliferation, inhibit apoptosis, and regulate inflammation through VEGF and PTEN pathways. They also include proteins involved in metabolic processes like fructose and galactose metabolism and glycolysis, explaining their significant impact on endothelial cell and keratinocyte proliferation and viability [79].

Adipose-derived stem cells (ADSCs) are also promising sources of EVs due to the abundance of adipose tissue and the relatively simple extraction procedure. Numerous trials have demonstrated their potential in enhancing wound healing [60,79,90]. ADSC-EVs partake in every phase of wound healing, including the regulation of inflammation, angiogenesis, cell proliferation, and ECM remodeling. Their specific cargo, such as miR-126-3p, miR-125a-3p, and miR-210, are vital for angiogenic processes, along with proteins inducing the Wnt/β-catenin axis and growth factors [91]. However, differences in the metabolic functions of adipose tissue among individuals pose a significant challenge for the widespread use of ADSC-derived EVs [92].

Comparing the two most commonly used groups of stem-cell-derived EVs, significant differences have been exhibited. Deep sequencing of the small RNA profiles of these EVs revealed that while their RNA compositions are similar, their tRNA contents differ. ADSC-EVs are primarily associated with angiogenesis, promoting endothelial cell migration and angiogenesis via the HIF-1 signaling pathway. In contrast, BMSC-EVs are linked to proliferative processes, showing a greater effect on cell proliferation and viability [79].

Additionally, EXOs derived from placental mesenchymal stem cells are recognized for their robust secretion of cytokines and chemokines involved in various signaling pathways, making them advantageous for promoting angiogenesis. Placentas are abundant and easily accessible sources of mesenchymal stem cells (PMSCs), such as human amniotic epithelial cells (hAECs) [93] and human umbilical cord mesenchymal stem cells (hUC-MSCs), which exhibit stability and immunogenicity and can transport proteins and growth factors essential for wound healing [94].

Human umbilical cord mesenchymal stem cells (UCMSCs) have demonstrated potential in treating different wound types using targeted injection methods. In research, peri-wound injection of UCMSCs in diabetic skin wounds increased M2-macrophages and decreased M1-macrophage infiltration in vitro, while promoting new capillary formation and improving wound healing in vivo through the MiR-let-7b pathway, specifically via let-7b/TLR4 and TLR4/NF-κB/STAT3/AKT signaling. In a rat model of third-degree burns, UCMSC treatment through tail vein injection significantly reduced total white blood cells, neutrophils, and macrophages, thereby decreasing inflammation in both in vitro and in vivo settings [41].

Not only are mesenchymal stem cells under investigation. Positive outcomes were registered in a study considering EXOs derived from fibroblast cells. Human fetal skin cells were cultured to produce EXOs, which were applied to Wistar rats. Histopathological analysis of skin biopsies revealed that the high-dose EXOs group exhibited faster healing and collagen deposition compared to controls. Both EXO groups demonstrated significantly improved healing by day 12 [90].

Another promising potential in wound healing is offered by plant EXO-like nanovesicles, which overcome challenges associated with synthetic nanoparticles such as immunogenicity and cytotoxicity. They are non-immunogenic and possess efficient uptake capabilities, making them increasingly apparent as emerging therapeutics and drug delivery nanoplatforms. Wheat-derived exosome-like nanovesicles promote skin regeneration through dose-dependent proliferative and migratory effects on epithelial, endothelial, and dermal fibroblasts, significantly elevating collagen type I mRNA levels [91].

Furthermore, EVs can be derived from biofluids such as platelet-rich plasma (PRP). A recent study aimed at investigating the protective effect of PRP-Exosomes (PRP-EXOs) on fibroblasts in a high-glucose environment in vitro, and their therapeutic potential in a diabetic rat model in vivo, found that PRP-EXOs protect fibroblasts from high-glucose-induced oxidative stress and promote diabetic wound healing by activating the PDGF-BB-induced JAK2/STAT3 signaling pathway. Although research has primarily focused on EXOs, it has also been proven that PRP-derived microvesicles can play a crucial role in wound healing. An in vitro scratch assay demonstrated that isolated PRP-derived MVs can stimulate human keratinocyte migration effectively, similar to platelet-rich plasma (PRP) and activated PRP, suggesting that the positive effects of PRP on wound healing might be mediated by EVs [95]. Similar thriving results of MVs derived from PRP were observed in a recent study conducted in vivo on Wistar rats. Imam et al. observed a decrease in levels of TGF-β, which is involved in fibrosis development in chronic inflammation. Blocking agents for TGF-β and CTGF show mixed results in scar prevention, but MVs offer potential. The immune-staining intensity of tumor necrosis factor-alpha was dramatically reversed in the MVs group compared with the burn group, whereas that of connective tissue growth factor, collagen I, and III was significantly reduced in both groups [93]

Considering the latest studies and methods of administration of EVs, the most common method is local injection. Topical application is slightly less common, and the rarest method is intravenous administration. However, one study reported the superiority of intravenous administration compared to subcutaneous injection [84].

Local injection involves administering EVs directly into or around wounds, typically subcutaneously, to enhance the healing process. The injections are generally made in the dermal layer, allowing saves to directly influence cell behavior and improve the wound microenvironment. Studies have shown that local injection of sEVs, such as adult MSC-derived sEVs or hucMSC-sEVs, can significantly promote wound healing, particularly in chronic diabetic wounds, through mechanisms like enhanced angiogenesis.

Topical application methods primarily involve stem-cell-derived EVs. One method is encapsulation in hydrogel, which Yang et al. demonstrated to be beneficial for full-thickness cutaneous wound healing in vivo. This method significantly accelerated wound closure, increased the expression of CD31, Ki67, VEGF, and TGFβ-1, and enhanced granulation tissue regeneration. Interestingly, hydrogel systems can be incorporated with other therapeutic components, such as functionalized gold nanorods (AuNRs) and M2 macrophage-derived exosomes (M2-EXOs). In experimental animal models of oral mucosa ulceration and full-thickness skin defects, the hydrogel demonstrated accelerated wound closure facilitated by M2-EXOs release [53].

Hydrogel microneedle (MN) patches, created using various techniques, are gaining attention in tissue engineering due to their biocompatibility, minimal invasiveness, prolonged drug retention, and high loading efficiency. M2-EXOs offer anti-inflammatory benefits but face issues like short lifespans and instability. A novel double-layer microneedle-based dressing (MEs@PMN) has been developed to address these challenges, encapsulating MEs in the needle tips and polydopamine nanoparticles in the backing layer. This system suppresses inflammation and enhances angiogenesis at the wound site [96].

Moreover, scaffolds seem to be a popular method of topical administration. They play a critical role in wound healing by providing structural stability and a conducive environment for cellular regeneration, mimicking the native tissue’s functionality. These biomaterial-based structures support the healing process by aiding cellular adhesion, proliferation, and differentiation. For instance, in one study, a polysaccharide-based fluorinated ethylene propylene (FEP) scaffold was developed to sustain EXO functionality. This scaffold exhibited multifunctional properties, including injectability, self-healing, adhesion, antimicrobial, hemostatic, and UV-shielding capabilities [97].

The administration method also depends on the source of cells from which the EVs are derived. For instance, EVs derived from bone marrow stem cells (BMSCs) are administered in most research by injections, showing improvements in inflammation, collagen deposition, and wound healing. A study by Lu et al. demonstrated the effectiveness of subcutaneous injection of EVs derived from preconditioned BMSCs in reducing inflammation, promoting collagen synthesis, and enhancing angiogenesis, thereby significantly improving wound healing outcomes [98]. Nonetheless, BMSCs and their EVs can also be delivered via scaffolds, which have been suggested to improve collagen deposition. The study showed that delivery of activated murine BMSC in a collagen scaffold enhances wound healing in a diabetic mouse model [99].

When it comes to adipose-derived stem cells (ADSCs) and their EVs, they are administered mainly through hydrogels or 3D-printed scaffolds, which have been shown to promote neovascularization, re-epithelialization, and modulation of inflammation. Researchers have explored combining ADSC-EVs with hyaluronic acid (HA) and hydrogels, which serve as EXOs immobilizers and wound dressings. These combinations decrease inflammatory cell infiltration and enhance therapeutic effects by providing a supportive environment for sustained ADSC-EV release. Moreover, thermoresponsive and thermosensitive hydrogels, such as Pluronic F-127 and GelMA-DOPA, have shown effectiveness in reducing inflammation and promoting tissue regeneration. Scaffolding materials like decellularized cardiac tissue, human acellular amniotic membrane (hAAM), and nanofibers further enhance ADSC-EV regenerative capacity and immunomodulatory effects, aiding in reducing inflammation and promoting wound closure [92,100].

EVs derived from biofluids, like platelet-rich plasma (PRP), can be administered in various ways. Local administration of PRP-EVs faces challenges due to their pro-coagulant activity. A recent study highlighted that PRP-EVs from resting platelets have milder pro-coagulant properties compared to those from thrombin-activated platelets. To reduce this activity for effective local administration, PRP-EVs derived from specific triggers are necessary. Additionally, PRP-EVs suffer from low retention rates and short treatment effects. Another study demonstrated that incorporating PRP-EVs into thermosensitive hydrogels enhances their local retention in tissues [101,102].

Platelet EXO products can also be combined with the fibrin sealant TISSEEL, enriched with TGF-β, to promote cell proliferation, migration, tube formation, and skin organoid formation in vitro, providing a cell-free, accessible, and cost-effective regenerative treatment for wound healing. This combination is administered in the form of a biogel, with PEP dissolved in a fibrinolysis inhibitor solution from the TISSEEL kit and mixed with a thrombin solution for local administration [103].

Possible complications in EV therapies are not clear and demand careful research. In vitro studies consistently show that ADSCs can enhance tumor cell proliferation and invasiveness across various cancer types, yet clinical reports do not indicate increased tumor incidence or recurrence post-ADSC therapy. It remains uncertain whether ASCs merely promote tumor tropism in pre-existing cancerous cells or potentially induce malignant transformation in normal cells [104]. To address these concerns, initiatives like the General Registry for Autologous Fat Graft (GRAFT) and ongoing clinical trials are pivotal [105]. When it comes to diabetic wound healing, miR-182-5p has been identified as a key player in EXs, promoting proliferation, migration, and angiogenesis. However, miR-182 is also known to facilitate epithelial–mesenchymal transition, invasion, and migration in breast cancer and hepatocellular carcinoma. This dual role indicates a potential cancer risk, as miR-182-5p could inadvertently promote tumor growth and angiogenesis while aiding wound healing [23]. 

**Table 3:** Clinical studies showing EVs potential in wound healing.

Year	Source of EVs	Experimental Model	Result
2023	MVs derived from mesenchymal stem cells and platelet-rich plasma	In vivo on animals	MVs derived from mesenchymal stem cells and PRP may improve burn wound healing via regulating scar formation and antioxidant mechanism [23]
2023	EXOs derived from endothelial progenitor cells	In vitro	The data showed that EPCs-EXOs promoted the proliferation and migration, while inhibited apoptosis of HaCaTs challenged by HG via elevating miR-182-5p expression level in vitro [98]
2023	EXOs derived from fibroblast cells	In vivo on animals	The results showed that the utilization of fibroblast-EXOs significantly promoted cutaneous wound healing in a rat full-thickness skin ulcer model [106]
2023	PRP-EXOs	In vivo and in vitro	PRP-EXOs can stimulate fibroblast functions and accelerate diabetic wound healing [23]
2021	dermal fibroblast-EXOs	In vitro and in vivo on animals	This research discovered that subcutaneous injections of DF-Ex could significantly promote re-epithelialization, collagen deposition, skin cell proliferation, and angiogenesis and inhibit inflammation to accelerate diabetic cutaneous wound healing [88]
2021	platelets exosome product	In vivo and in vitro	In vitro, PEP significantly promoted cell proliferation, migration, and tube formation, as well as skin organoid formation [103]
2020	PRMVs	In vitro	The research found that PRP pro-healing effects were fully replicable by PLT-MVs, suggesting a key role of MVs in the healing process and a possible clinical use as an alternative to PRP [95]
2018	BMSC-EVs	In vivo and in vitro on animals	The study concludes that murine ADSC and BMSC are equally effective in enhancing diabetic wound healing, and human diabetic ADSC is as effective as non-diabetic ADSC [99]

## 7. Conclusions

Due to their significant impact on various physiological and pathological processes, EVs deserve attention as well as continuous research. They influence cell proliferation, migration, and differentiation along with angiogenesis, stress responses, inflammation, ECM formation, and regeneration. Their promising potential lies in their minimal toxicity accompanied by low immunogenicity, which makes them perfect for drug delivery systems, superior to synthetic carriers. Separate pathways of EXO and MV synthesis confer their differences in size, marker proteins, and cargo. EXOs were proven to mitigate inflammation, promote angiogenesis, reduce ROS production, preserve homeostasis, and enhance cellular autophagy. Their implementation was found to be promising in neurological, cardiological, ophthalmologic, and dermatological diseases among others. MVs promote cell migration, proliferation, and vascularization. Furthermore, they take part in inflammatory processes and wound healing. Research has proven their role in both liver and kidney regeneration. Many clinical trials are currently being performed, aiming to establish the application of EXOs and MVs as drug delivery carriers, therapeutic agents, or biomarkers. Nevertheless, their optimal use remains a challenge in large-scale production, isolation, and drug loading due to technological limitations. However, new promising potential approaches for the technological processes of their utilization give hope for large-scale implementation in the future. These advances may offer a novel, superior treatment; therefore, there is still an ongoing need for further research regarding EXO and MV regenerative impacts and their possible application in treating diseases, along with establishing possible side effects and other disadvantages of this method. 

## Figures and Tables

**Table 1:** Mechanism of EXOs in wound healing.

Mechanism	Active Component	Signaling Pathways
Macrophage polarization M1 M2	miRNA + snRNA	TGFβ signaling [25]
	miRNA let-7b	inhibition of NF-κB pathway [30]
	MFG-E8	STAT3 pathway [32]
	miR-203a-3p	STAT3 pathway [39]
	miR-146a-5p	inhibition of NF-κB pathway [40]
	miR-181c	inhibition of NF-κB pathway [41]
The promotion of proliferation state	bFGF, PDGFBB, and TGF-β	PI3K/AKT + Erk pathway [21]
	-	VEGF signaling pathway [42,43]
	miR-126-3p	PI3K/AKT and Erk pathways [44]
	lncRNA MALAT1	activation of Wnt/β-catenin pathway [46]
Inhibition of scar formation	lncRNA MALAT1	Wnt/β-catenin pathway [46]
	14-3-3ζ	Wnt/β-catenin pathway [47]

**Table 2:** Mechanism of MVs in wound healing.

Mechanism	Active Component	Signaling Pathways
Macrophage polarization M1 M2		STAT signaling axis [52]
The promotion of proliferation state	miRNA-145 and miRNA-132	S1P signaling pathway [55]
	VEGF, FGF-2, and PDGF	PI3/AKT + Erk pathway [58]
	miR-16-5p	p38/MARK pathway [61]
	Wnt4	Wnt/β-catenin pathway [79]
Inhibition of scar formation	14–3-3ζ protein	Wnt/β-catenin pathway [64]

## Data Availability

No new data were created or analyzed in this study.
